# Implementation of Integrated Primary Care for Patients with Diabetes and Hypertension: A Case from Slovenia

**DOI:** 10.5334/ijic.5637

**Published:** 2021-09-28

**Authors:** Zalika Klemenc-Ketis, Nataša Stojnić, Črt Zavrnik, Nina Ružić Gorenjec, Katrien Danhieux, Majda Mori Lukančič, Antonija Poplas Susič

**Affiliations:** 1 Ljubljana Community Health Centre, Metelkova 9, 1000 Ljubljana, Slovenia; 2 Department of Family Medicine, Faculty of Medicine, University of Ljubljana, Poljanski nasip 58, 1000 Ljubljana, Slovenia; 3 Department of Family Medicine, Faculty of Medicine, University of Maribor, Taborska 8, 2000 Maribor, Slovenia; 4 Institute for Biostatistics and Medical Informatics, Faculty of Medicine, University of Ljubljana, Vrazov trg 2, 1000 Ljubljana, Slovenia; 5 Department of Primary & Interdisciplinary Care Antwerp, University of Antwerp, Belgium

**Keywords:** delivery of health care, integrated, primary care, self-care

## Abstract

**Introduction:**

Research on models of integrated health care for hypertension and diabetes is one of the priority issues in the world. There is a lack of knowledge about how integrated care is implemented in practice. Our study assessed its implementation in six areas: identification of patients, treatment, health education, self-management support, structured collaboration and organisation of care.

**Methods:**

This was a mixed methods study based on a triangulation method using quantitative and qualitative data. It took place in different types of primary health care organisations, in one urban and two rural regions of Slovenia. The main instrument for data collection was the Integrated Care Package (ICP) Grid, assessed through four methods: 1) a document analysis (of a current health policy and available protocols; 2) observation of the infrastructure of health centres, organisation of work, patient flow, interaction of patients with health professionals; 3) interview with key informants and 4) review of medical documentation of selected patients.

**Results:**

The implementation of the integrated care in Slovenia was assessed with the overall ICP score of 3.7 points (out of 5 possible points). The element Identification was almost fully implemented, while the element Self-management support was weakly implemented.

**Discussion:**

The implementation of the integrated care of patients with diabetes and/or hypertension in Slovenian primary health care organisations achieved high levels of implementation. However, some week points were identified.

**Conclusion:**

Integrated care of the chronic patients in Slovenia is already provided at high levels, but the area of self-management support could be improved.

## Introduction

Although there is no single definition of integrated care, it can be described as a coherent and coordinated set of services planned, managed and offered to individual service users by a number of organisations and a range of cooperating professionals and informal carers [1].

Diabetes and hypertension have been prioritised in global and national action plans because of the high burden of disease, the availability of cost-effective interventions and a strong consensus on their relevance, effectiveness and necessity [2,3].

The integrated care package for patients with diabetes type 2 (T2D) and hypertension (HT) consists of six components: (a) identification of people with HT and/or T2D and subsequent (b) treatment in primary care, (c) health education, and (d) self-management support by patients and caregivers, (e) collaboration between caregivers, and f) organisation of care [4].

Research on models of integrated health care for hypertension and diabetes has been identified as one of the priority issues because it can provide the solution to several long-standing problems in the health care system and public health approach to both diseases, such as the lack of continuity of care, the fragmentation of medical care/treatment processes and the quality of patient education [5]. In general, there is evidence that the process and outcomes of integrated care improve the quality of care and its outcomes [6,7,8], but there are still significant gaps, such as the combination of diseases, the combination of elements, and implementation and scale-up [5].

Slovenia is a Central European country of about two million inhabitants. Its national health system can be described as a combination of the Beveridge and Bismarck models with the main principles of universal coverage, solidarity, fairness in financing, non-profitability and equity in access for all groups of population. All permanent residents of Slovenia are included in compulsory health insurance at the National Insurance Institute; almost 95% of population has in addition a voluntary complementary insurance. The state’s task is to prepare and set up the primary health care network of family physicians, primary gynaecologists, dentists and paediatricians, who work either in a health centre together with other family physician working teams, or as an independent contractor (a physician who has a private family practice but has a contract with the National Insurance Institute) [9,10].

In 2011, the Slovenian government invested in scaling-up of the management of chronic disease patients in family medicine [11]. This process ensured standardized the approach to screening, patients’ management, quality assurance and regular reporting; improved the quality of care; enabled an integrated, standardized, and person-centred approach to patients; and enabled a division of workload among all members of a team according to their competencies. It includes also the use of the standardised protocols for diagnosis and treatment of diabetes and hypertension, health education, and guidelines on collaboration within the care team and between different providers e.g. health education centres in region, municipalities, clinical specialists on the secondary/tertiary care levels, social workers, and patient associations, which also provide health education and self-management support, etc. The protocols are continuously monitored and adapted according to new evidences by a steering group of the Ministry of Health, consisting of professionals in the required fields. This integrated way of managing patients is financed through the Health Insurance Institute of Slovenia [9].

Each family medicine team works with the electronic system (there are six different systems in Slovenia) [12]. The protocols are not integrated directly into the electronic system, but their use is obligatory. The protocols consist of a clear description of the tasks that need to be done at primary care level for preventive activities and managing chronic patients [13].

The aim of this study is to evaluate the implementation of integrated care for patients with diabetes and hypertension in Slovenian primary health care and to detect possible differences between urban and rural regions of Slovenia.

## Research Methods

### Study type and settings

This was a mixed methods study based on a triangulation method using quantitative and qualitative data. It was conducted in primary health care organisations from rural and urban areas. This study was a part of a larger project: Scaling up an integrated diabetes and hypertension care package for vulnerable people at risk in Cambodia, Slovenia and Belgium (SCUBY) [4]. SCUBY is an international research project co-funded by the EU under the H2020 – Health programme (H2020-SC1) with contract number 825432 – SCUBY. In Slovenia, 10 family medicine teams participated, eight from an urban health centre (central Slovenia) and two from two rural health centres (one from the northern region and one from the eastern region of Slovenia). The sampling of health organisations was purposive. We wanted to include family medicine teams from rural and urban parts of Slovenia, as well as from large community health care centres with many family practice teams and from smaller (single-handed) practices (independent private contractors. Within larger health care centres, the sampling of the family practice teams was random.

They National Ethics Committee approved the study (No. 0120-219/2019/4).

### Participants

In each primary health care organisation, an extended family medicine team involved in the management of patients with diabetes and/or hypertension participated. Each team involved a family physician, a practice nurse, a registered nurse, a registered nurse working in a health education centre and a community nurse. We included those primary health care facilities where only one family physician works with his/her team, and those (i.e. health care centres) where more family physicians work with their teams. If more teams worked in one health care facility, we included them purposively so all different profiles were included.

Registered nurses work in an extended field of practise involving the diagnosis, prescription and treatment of diseases in specific settings [14]. Practice nurse is a secondary school degree nurse whose tasks in the family practice teams are administration, appointments, and clinical work (taking care of wounds, point-to-care measurements etc.) [15]. A community nurses take care of patients at home. They provide healthcare to new-born babies and their mothers, and preventive health and social care to individuals and families in their home environment. Under the supervision of the family physician and at their request, a community nurse can also provide curative healthcare to patients in their homes [16].

All participants gave the oral informed consent to participate in the study.

### Instruments

The main instrument for data collection was the ICP (Integrated Care Package) Grid. It was developed among the members of the project team from all three participating countries in the following subsequent steps. In the first step, they reviewed the available tools that could suit to the purpose of this study and identified two possible tools: the Assessment of Chronic Illness Care form (ACIC) [17,18] and the Assessment of Innovative Care for Chronic Disease framework tool (ICCC) [19]. These two tools have already been validated and both of them are widely used in high and low income settings to assess the degree of implementation of integrated chronic care. However, they are not disease specific and were therefore tailored to diabetes and hypertension by adding specific questions about these diseases. All questions were tailored separately for diabetes and hypertension, by adding these word to the questions.

In the second step, ICP Grid was tested for understandability and feasibility among professionals (team members managing patients with diabetes and hypertension) in each participating country. As a result, some changes were made. The third step was a translation to the national languages. This was done through a backward and forward process [20]. Finally, the tool (in national languages) was tested for understandability and feasibility among the users (members of the family practice team) in each country and the final changes were made.

The ICP-Grid consists of 6 elements: Identification (8 items), Treatment (15 items), Health Education (8 items), Self-Management support (13 items), Structured Collaboration (10 items), and Care Organisation (6 items) (Appendix 1).

Items of the ICP grid were answered on a scale from 0 to 5 (0 – no implementation of the ICP, 1 – little implementation of the ICP, 2–3 – moderate implementation of the ICP, 4 – almost complete implementation of the ICP, 5 – full implementation of the ICP). For each of the items on the ICP Grid, we defined what individual score meant/described (see Appendix 1).

### Collection of data

ICP Grid was used for the collection of data. It enables assessment of multiple resources to select the final answer. We used the following multiple data resources: a document analysis (of a current health policy and available protocols); an observation at the facility (of the health facility infrastructure, organisation of work, patient flow, interactions of patients with health care workers); interviews with key informants (team members working with chronic patients); and inspection of documentation at the health facility (management books, patient registries, random patient files check when needed).

Taking all the data resources into account, two researchers filled in the ICP Grid separately. Later, they compared their scores and arrived to a consensus – a single score for each ICP Grid item. If the score was not the same and they could not achieve consensus, a previously appointed supervisor who was an expert in the area of family medicine and in the qualitative research methodology was included to help to achieve consensus.

The same two researchers assessed all health facilities. In each facility, the same data sources were used.

### Analysis of the data

For each primary health care organisation, the score for each ICP grid element was calculated as the mean of the items and standard deviations were reported. The overall ICP score of a health care institution was assessed as a mean of scores for separate ICP Grid elements. The scores for the regions were calculated as the mean value of the scores for the corresponding health care organisations, while the scores for the country as a whole were calculated as means of the scores for the regions. To compare the elements between regions (urban/rural), t-tests for independent samples were used and a p-value of less than 0.05 was considered statistically significant.

## Results

**Figure 1:**
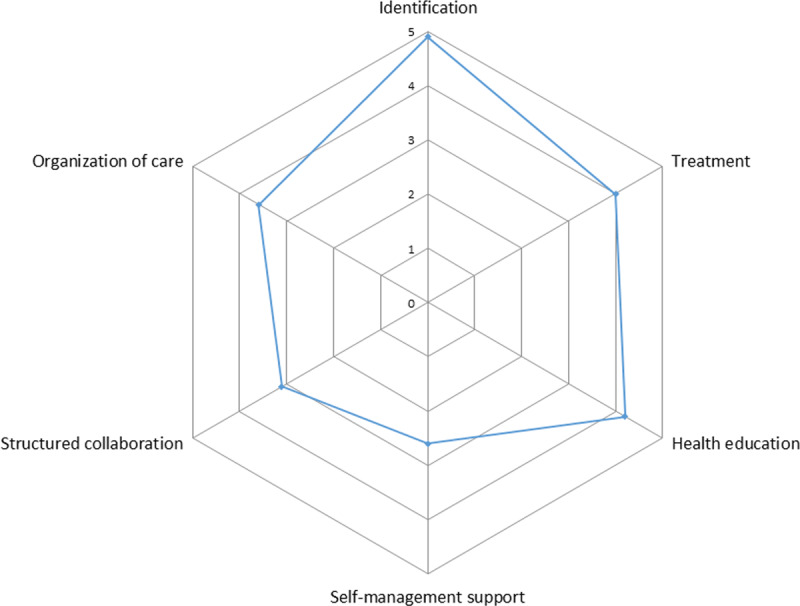
Graphic presentation of the ICP Grid scores for Slovenia.

**Figure 2:**
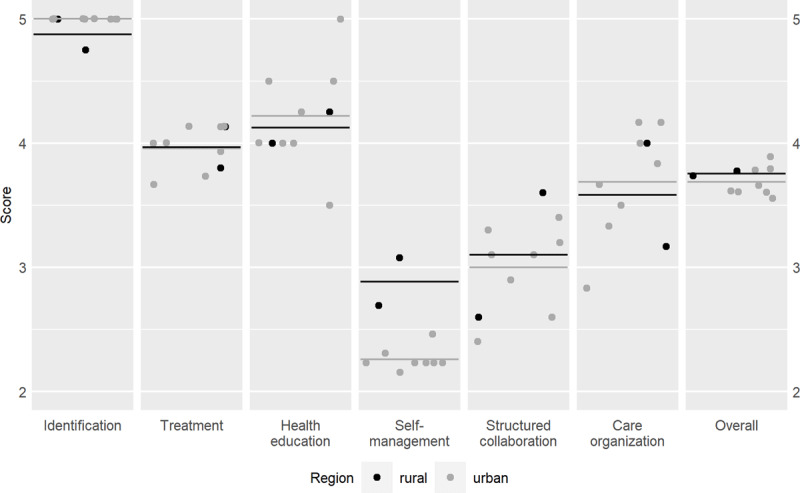
Graphical presentation of the ICP Grid scores for individual health care organisation and for regions in Slovenia.

In all participating health organisations, the implementation of the integrated care was assessed with overall ICP scores between 3.6 and 3.9 points (Appendix 2), which resulted in the overall ICP score for Slovenia of 3.7 points (out of 5 possible points) (***Table 1***). In the ICP scores for Slovenia, two elements stood out: Identification received the highest rating for implementation (4.9 points), while Self-management Support received a low rating (2.6 points) (***Table 1***), also graphically presented in ***Figure 1***. Scores of ICP grid for individual health care organisation and for regions are graphically summarised in ***Figure 2*** (the corresponding values are in ***Table 1*** and Appendix 2).

**Table 1:** Evaluated implementation of integrated care for patients with diabetes and/or hypertension in Slovenia and separately for urban and rural regions.


ICP GRID ELEMENT	SLOVENIA	RURAL REGION MEAN (MIN, MAX)	URBAN REGION MEAN (MIN, MAX)

E1 – Identification	4.9	4.9 (4.8, 5.0)	5.0 (5.0, 5.0)

E2 – Treatment	4.0	4.0 (3.8, 4.1)	4.0 (3.7, 4.1)

E3 – Health Education	4.2	4.1 (4.0, 4.3)	4.2 (3.5, 5.0)

E4 – Self-management Support	2.6	2.9 (2.7, 3.1)	2.3 (2.2, 2.5)

E5 – Structured collaboration	3.1	3.1 (2.6, 3.6)	3.0 (2.4, 3.4)

E6 – Care organization	3.6	3.6 (3.2, 4.0)	3.7 (2.8, 4.2)

Overall	3.7	3.8 (3.7, 3.8)	3.7 (3.6, 3.9)


The variability within elements Self-management Support, Structured Collaboration and Care Organisation was greater than within other elements (see Appendix 2). For all elements but Self-management Support, assessments were similar in rural and urban regions, while the difference in Self-management Support on our sample was 0.6 (2.9 points in rural and 2.3 points in urban regions) (***Table 1***), which we consider important as it would represent 12% on a 0–100% scale. However, none of the differences in the elements between regions were statistically significant (smallest p was equal to 0.182), but the sample sizes of groups were small (2 in rural, 8 in urban).

### Element Identification

For this ICP Grid element, score for Slovenia was 4.9 (out of 5.0) (***Table 1***). Participants pointed out that population identification or screening was performed equally by all health care teams in both urban and rural regions. They also expressed that screening was performed according to a protocol that dictates all persons over 30 to be screened for risk factors and risk for developing T2D and HT. It was found that appropriate equipment exists and health care team is competent to screen and follow patients.

### Element Treatment

For this ICP Grid element, score for Slovenia was 4.0 (out of 5.0) (***Table 1***). Participants pointed out that family physicians are competent to diagnose and treat patients. It was found that there was a good collaboration with diabetologists, which results in a fluent referral system. Physicians expressed that the updated guidelines were available to them but they miss pop-up windows with a short summary of the important results integrated to the electronic medical record.

### Element Health Education

For this ICP Grid element, score for Slovenia was 4.2 (out of 5.0) (***Table 1***). Participants pointed out that patient groups are provided with structured education. They also reported that vulnerable groups sometimes do not respond to invitations and nurses want fixed dates for group meetings. Participants also expressed that they felt qualified for health education. However, they said that there are too few health education or counselling materials and lack of video material and didactically designed material, but pointed out that the existing health educational material is exemplarily prepared.

### Element Self-management Support

For this ICP Grid element, score for Slovenia was 2.6 (out of 5.0) (***Table 1***). Participants pointed out that self-management training is available for patients. Also, an important obstacle in Slovenia was identified: patients with diabetes that are on diet or on oral therapy have to purchase glucometer and associated slips on their own which results in few patients having and using these items. It was found that relatives could be involved more in the self-management process (such as in case of patients with dementia). Participants also expressed that even if the self-management plan is not structured, many times a written reminder, scheme or plan is given to the patient.

### Structured collaboration

For this ICP Grid element, score for Slovenia was 3.1 (***Table 1***). Qualitative data pointed out that in Slovenia there is no formal care coordinator. The participants pointed out that referrals and medical reports are systematically organized but often not entered to the electronic records, there are IT problems with the transmission of results and the registered nurses are not authorized to see them.

### Care organization

For this ICP Grid element, score for Slovenia was 3.6 points (***Table 1***). Qualitative data pointed out that the participants were satisfied with the introduction of improvements in care organization, but they do not get the feedback about the evaluation of the implemented changes.

## Discussion

The results of this study showed that the implementation of the integrated care of patients with diabetes and/or hypertension in Slovenian primary health care organisations has achieved a high level. However, some room for improvement was identified. The element Identification was implemented at the highest possible level, while the element Self-management support was only weakly implemented. In rural areas, the Self-management Support element was implemented at a slightly higher level than in urban areas but the reason for that did not arise in the qualitative part of our analysis nor was our primary goal. We believe that these levels of implementation are a consequence of an earlier mentioned national project [11], which implemented regular screening for most frequent chronic diseases for a population of 30 years and older, and the introduction of a protocol for chronic patients management, both in 2011. Importantly, the project did not address an area of self-management support.

Also other studies (see below) reported that integrated care of chronic patients was in general well implemented, with several week points, although direct comparisons were not possible due to the different research methodology. A study from Denmark showed a partial level of implementation of integrated care [21], and similar results were found in a meta-review [6]. Integrated care for patients with diabetes and/or hypertension exists in many countries, but only some of its elements are implemented in practice [5].

Treatment and Health Education elements were shown to be implemented at high levels. These elements are very well defined in protocols for family practice teams working with chronic patients [9,12,22]. These protocols have been harmonised between primary and secondary care and between the different health care professions involved in the care of the chronically ill [13]. They are used in all primary care practices in Slovenia, and this is probably the reason for the high level of implementation of these ICP elements in Slovenia, regardless of the region.

ICP elements that showed lower implementation levels were the organisation of care, structured cooperation and support for self-management. The Care Organisation element describes quality assessment and improvement. This field is currently in development in Slovenia. Several quality indicators have been introduced, but continuous quality improvement with feedback from practice, suggestions for improvement and regular evaluation of quality improvement has not yet been implemented [23]. Therefore, low scores for this element are not surprising. Our study showed that structured collaboration in Slovenia could be improved, as shown in previous studies [15,24]. Most of the communication problems are between different professionals, especially due to the fact that there is no role of the care coordinator established within the system [15]. For structured collaboration, no defined specific protocol or services exist, approved by the Health Insurance Institute. Namely, this Institute determines a set of services allowed and paid for at the primary care in Slovenia. If such protocols at national level, approved by the professionals and Slovenian Health Insurance Institute, would be developed, they would improve the interprofessional collaboration and therefore a quality of patient care.

Self-management support was rated as the one with the lowest degree of implementation of all elements. Studies show that self-management is the key to successful management in chronic patients [25,26]. This part of integrated care in Slovenia has the greatest potential for improvement. At present, patients receive instructions on self-care during consultations with the family practice team. They can enrol in various self-support groups, but neither individual self-management support nor national guidelines on self-management support have been implemented yet. The problem is also the availability of the gadgets for self-monitoring of the disease. In Slovenia, attempts on how to help patients to self-manage their chronic diseases with the use of IT technology were done almost 10 years ago [27], but there is no implementation in clinical practice at the primary care level yet. Since the burden of chronic diseases is increasing and health care professionals will not be able to consult all patients in person in the future [28], that kind of self-supporting is becoming important worldwide and the COVID-19 situation is stimulating this process very much [29]. For those elements that scored lower, there was greater variability in item scores. It is likely that each health care organisation has its own discretion in organising care in these aspects of integrated care. This could indicate that these areas of integrated care in Slovenia are insufficiently organised due to the lack of national guidelines and recommendations.

In general, there were no significant differences between rural and urban areas, which suggest that the level of implementation of integrated care is quite similar in rural and urban areas. This contrasts with studies in other countries, which show that integrated care is not implemented or managed as well in both rural and urban areas [30,31]. This suggests that there is a viable model of integrated primary health care in Slovenia for all regions, both rural and urban. However, there was an important difference between regions on our sample in the implementation of the Self-management Support: it was 0.6 points higher in rural regions on a 0–5 scale; this would represent 12% on a 0–100% scale. Nonetheless, this cannot be generalised to all Slovenian regions as the difference was not statistically significant, possibly due to the small sample sizes of groups (2 in rural, 8 in urban) which is a limitation of our study.

One possible explanation for the urban/rural differences could be that people in rural areas of the country are more connected because they live in smaller communities, know each other better and therefore help each other more. For rural areas, a more detailed insight into good practice in self-management support is needed so that the findings can be applied in urban areas. One of the independent urban contractors had a slightly better assessment of self-management support, which may indicate that self-management support is easier to organise in smaller organisations and depends more on the interests of the care provider than on the general orientation in the country.

Primary care in Slovenia needs improved measures to support self-management for the chronically ill patients. Patients expect health-care professionals to fulfil a comprehensive role, providing education and relational support [32]. However, family members and appropriately trained patients can also provide support [32], and this should be taken into account when exploring the possibilities of scaling-up. Family-based care interventions have the potential to improve the health and well-being of people with chronic diseases [33]. One possible method is the introduction of a care manager. This is a designated professional with a supervisory role in coordinating care in an ongoing relationship with the patient. The qualifications of a care manager may vary across different models of integrated care management. Depending on the model, a care manager may be based in a single practice for primary care or work with several medical practices [30]. Another option are lay caregivers, who are trained laypersons who assist patients in the community [34]. This could enable the down-step care from the primary care level to the patients themselves. Through that process, patients receive competencies for self-management and their active role in the management of their disease development. The use of tele monitoring and web-based interventions, which have been shown to be useful in diabetes patients for better self-management of their disease [35], is also promising.

The strengths of our study are that all researchers are working in primary care and are very familiar with the context. In addition, we used a strong framework based on two validated and widely used scales for assessing chronic care, which were adapted to the needs of the study. This study has some methodological considerations. We decided to use a mixed methodology based on a triangulation method using quantitative and qualitative data, as we wanted to detect differences in the implementation of integrated care in primary health care organizations, for which we used ICP Grid as our measurement instrument. Even if the ICP Grid requires qualitative assessments (interviews etc.), its output is a score that allows the use of statistical techniques and is therefore in its nature quantitative.

We used the adapted tool to assess the implementation of integrated care, as we could not find existing tools specifically addressing diabetes and hypertension. Also, there is currently no common framework in the literature for integrated care of the chronically ill [36]. However, this instrument has been adapted for the specific purpose of our study and is based on two validated similar instruments. This could also be the strength of our methodology.

A recent scoping review showed that the following five components were the most frequently mentioned as components of integrated care: person-centred care, holistic needs assessment, integration and coordination of care, collaboration and self-management [37]. Another literature review pointed to 12 categories of integrated care, including self-management support, decision support and organisation of care [36]. Our six elements correspond to all these elements from the literature, which gives us confidence in the relevance of the tool used in our study. Some considerations are only regarding the element one (Identification). It only examines the readiness and competence of the facility, but does not take into account population coverage and screening capacity. This could lead to potentially higher scores of element one, which should affect the representativeness of the study.

Another limitation is that the included health care organisations came from only three out of ten health regions in Slovenia, all urban organisations were from the same region and there were only two rural organisations. Our sampling of the teams was not random, so there can also be some level of selection bias. But according to the implemented clinical pathways existing for management of chronic diseases in Slovenia, we presume that no substantial deviation from existed results would be found. Also, this study showed that the elements of the ICP grid that are managed by protocols (Identification, Treatment, Health Education) received high and consistent values in all regions studied. This gives us further confidence in the reliability of our results.

Another limitation is the bias of respondents, as respondents may be led to give desirable answers that do not always correspond to reality – this could lead to an overestimation of the scores. However, using a triangulation method (asking, observing, checking documentation) reduced the respondents’ bias.

There are some considerations regarding the calculation of the scores. The score for each ICP grid item was calculated as the mean value of the corresponding items, as this is part of the instrument and not of the data analysis. In this way, each item contributes equally to the evaluation of the element. As far as data analysis is concerned, we implicitly use weights, as we first calculate a mean for the urban and a mean for the rural region and then take their mean as the score for Slovenia, i.e. the two rural organisations receive a weight of 1/4 each and each of the eight urban organisations receives a weight of 1/16. In this way we take into account that the number of urban and rural organisations in the sample is not representative for Slovenia. It may be suggested that the organisations could be weighted differently, but it is important that the weights reflect the population and this is difficult to achieve. For example, weighting according to the number of patients could be problematic if the smaller of our two rural organisations were actually more representative of Slovenian rural areas than the larger one. That is why we have chosen the simpler version of weighting (as described above), as the results could in any case be biased.

## Conclusion

Integrated care of the chronic patients in Slovenia is already provided at high levels. However, there is a need for improvement, especially in the area of self-management support. National protocols for chronic diseases can lead to a high degree of implementation of health care. These protocols should be developed and implemented for each element of integrated care, probably also adapted contextually for different regions. Further studies in Slovenia should focus on the evaluation of facilitators and barriers to the implementation of integrated care, especially the elements that were found to be implemented at lower levels such as Self-management Support, and possible solutions. The tool developed in this study has proven to be a useful framework for assessing the implementation of integrated care for two major chronic diseases. Its further application in other countries will strengthen the evidence base for the implementation and expansion of integrated care. A comprehensive picture of the quality of the integrated care cannot be obtained without patient-reported indicators, so further studies should also use tools such as patient-reported outcomes and experiences.

## Additional Files

The additional files for this article can be found as follows:

10.5334/ijic.5637.s1 Appendix 1. ICP Grid.

10.5334/ijic.5637.s2 Appendix 2. Assessed implementation of integrated care for patients with diabetes and/or hypertension according to individual health care organisation.
