# Supplementary material for: Implementation of Integrated Primary Care for Patients with Diabetes and Hypertension: A Case from Slovenia
Source: Int J Integr Care. 2021 Sep 28;21(3):15. doi: 10.5334/ijic.5637 (PMC8485865; doi:10.5334/ijic.5637)
Supplement: Appendix 1. — ICP Grid. [file ijic-21-3-5637-s1.pdf]

Appendix 1: ICP Grid

**Name of unit tested:**						
**Name of researchers filling grid:**						
**Date of assessment:**						
**Integrated care package (ICP)**						
**ICP Element**	**Response**					
Element 1:<br>**Facility based identification of patients with HT and/or T2DM**<br>*Facility: place (health center, hospital, clinic, home, headquarter) where care is provided*	No or little implementation		Moderate implementation		Almost complete or full implementation	
1.1.a. To what extent, is **screening for DM** performed among patients at a visit? [PROCESS]	**0**<br><br>Not at all	**1**<br><br>Only on patient's initiative, not based upon HCW thinking of it = client-based	**2**<br><br>When diabetes symptoms or required by other conditions	**3**<br><br>When required by risk factors but not consistently done	**4**<br><br>Consistently done in a group of patients defined by risk factors (almost everyone).	**5**<br><br>Everyone who needs to be tested gets tested.
1.1.b. To what extent, is **screening for HT** performed among patients at a visit? [PROCESS]	0<br><br>Not at all	1<br><br>Only on patient's initiative, not based upon HCW thinking of it = client-based	2<br><br>When required by other condition or hypertension symptoms	3<br><br>When required by risk factors but not consistently done	4<br>Consistently done in a group of patients defined by risk factors (almost everyone).	5<br><br>Everyone who needs to be tested gets tested.
1.2.a. To what extent, are **equipment and materials** necessary for diagnosing patients for **DM** available at the facility? [STRUCTURE]	0<br><br>Not at all	1<br><br>Available but not functional	2<br><br>Partially equipped – some parts not functional	3<br><br>Equipped for a small number of patients	4<br><br>Fully equipped for almost everyone	5<br><br>Fully equipped for everyone
1.2.b. To what extent, are **equipment and materials** necessary for diagnosing patients for **HT** available at the facility? [STRUCTURE]	0<br><br>Not at all	1<br><br>Available but not functional	2<br><br>Partially equipped – some parts not functional	3<br><br>Equipped for a small number of patients	4<br><br>Fully equipped for almost everyone	5<br><br>Fully equipped for everyone

1.3.a. To what extent, are health care staff or service providers **competent** to perform **diagnosis for DM** at the facility? [STRUCTURE/EDUCATION]	0<br>Not at all	1<br>Know but cannot perform properly	2<br>Perform with guidance from others	3<br>Properly perform cannot interpret the results	4<br>Properly perform with limited interpretation of the results	5<br>Properly perform with clear interpretation of the results
1.3.b. To what extent are health care staff or service providers in charge **competent** to perform **diagnosis for HT** at the facility? [STRUCTURE/EDUCATION]	0<br>Not at all	1<br>Know but cannot perform properly	2<br>Perform with guidance from others	3<br>Properly performed cannot interpret the results	4<br>Properly performed with limited interpretation of the results	5<br>Properly performed with clear interpretation of the results
1.4.a. To what extent is the **follow-up of the patients after the screening, testing and diagnosis of DM** organised?	0<br>No follow up	1<br>Pt are referred for diagnosis/ therapy but no follow up	2<br>Follow up only on patient's initiative	3<br>Follow up on if positive/diagnosed and not follow up if negative/high risk	4<br>Follow up if positive and if negative/high risk	5<br>Care is organised in such a way there is a planning for every pt and they are called if non- attending
1.4.b. To what extent is the **follow-up of the patients after the screening, testing and diagnostic test results** of **HT** organised?	0<br>No follow up	1<br>Pt are referred for diagnosis/ therapy but no follow up	2<br>Follow up only on patient's initiative	3<br>Follow up on if positive/diagnosed and not follow up if negative/high risk	4<br>Follow up if positive and if negative/high risk	5<br>Care is organised in such a way there is a planning for every pt and they are called if non- attending
Element 2: **Treatment of DM and HT by primary care providers using standardized protocols**<br>*Primary care providers: first line of care providers (not including those at hospitals)*	No or little implementation		Moderate implementation		Almost complete or full implementation	
2.1.a. To what extent, are **written guidelines of care and treatment** accessible to primary care providers for DM? [STRUCTURE]	0<br>Not available at all	1<br>Some guidelines available, but not used in daily practice or difficult to access	2<br>Easy accessible guidelines but not recently updated and not encouraged	3<br>Easy accessible guidelines and recently updated or their use is encouraged	4<br>Recent updated guidelines available, and their use is encouraged through posters and other educational	5<br>Recent updated guidelines available and integrated in daily practice through reminders (pop-ups) in electronicmedical record tailored to the

					PROCESSs etc.	patient
2.1.b. To what extent, are **written guidelines of care and treatment** accessible to primary care providers for HT?	0<br><br>Not available at all	1<br><br>Some guidelines available, but not used in daily practice or difficult to access	2<br><br>Easy accessible guidelines but not recently updated and not encouraged	3<br><br>Easy accessible guidelines and recently updated or their use is encouraged	4<br><br>Recent updated guidelines available, and their use is encouraged through posters and other educational PROCESSs etc.	5<br><br>Recent updated guidelines available and integrated in daily practice through reminders (pop-ups) in electronicmedical record tailored to the patient
2.2.a. To what extent, are primary care providers in charge **competent to provide treatmen**t for patients with **DM**?<br>[STRUCTURE/EDUCATION]<br><br>* professional training = continued professional education	0<br>No knowledge at all	1<br>Have some non-pharmacological knowledge	2<br>Have non-pharmacological knowledge and skills	3<br>Have detailed knowledge and non pharmacological skills also basic pharmacological knowledge	4<br>Have all detailed knowledge also about pharmacological treatment	5<br>Have detailed knowledge and know how to treat complications
2.2.b. To what extent, are primary care providers in charge **competent to provide treatmen**t for patients with **HT**?<br>[STRUCTURE/EDUCATION]	0<br>No knowledge at all	1<br>Have some non-pharmacological knowledge	2<br>Have non-pharmacological knowledge and skills	3<br>Have detailed knowledge and non pharmacological skills also basic pharmacological knowledge	4<br>Have all detailed knowledge also about pharmacological treatment	5<br>Have detailed knowledge and know how to treat complications
2.3.a. To what extent are the **essential medications** for DM available in the primary care setting?<br>[STRUCTURE]	0<br><br>Not at all	1<br><br>Only some medications available	2<br><br>Most of the basic medications available, but stock out accure, not indicated in PC	3<br><br>Most of the basic medications always available and indicated	4<br><br>Also advanced medications available but not indicated in primary care	5<br><br>Fully accessible to all necessary and all advanced medications (availability and indication)
2.3.b. To what extent, are the **essential medications** for HT available in the primary care	0<br><br>Not at all	1<br><br>Only some	2<br><br>Most of the basic	3<br><br>Most of the basic	4<br><br>Also advanced	5<br><br>Fully accessible to all

setting?<br>[STRUCTURE]		medications available	medications available, but stock out accure	medications always available	medications available but not indicated in primary care	necessary and all advanced medications
2.4. To what extent, do primary care providers have necessary **laboratory access**?<br>[STRUCTURE]	0<br><br>Not at all	1<br><br>Limited testing items, difficult accessibility	2<br><br>All required testing items, difficult accessibility	3<br><br>Easy accessibility to limited testing items	4<br><br>Easy accessibility to almost required testing items	5<br><br>Easy accessibility to all required testing items
2.5.a. To what extent have primary care providers received **training for treating** DM?	0<br>Not at all	1<br>On the job training or when services started	2<br>Part of formal education to obtain certificate needed to do the job	3<br>as in 2 + sporadic extra trainings on the topic	4<br>As in 2 + systematically extra trainings on the topic	5<br>As in 2 + systematically extra obligatory trainings on the topic, with innovative methods
2.5.b. To what extent have primary care providers received **training for treating** HT?	0<br>Not at all	1<br>On the job training or when services started	2<br>Part of formal education to obtain certificate needed to do the job	3<br>as in 2 + sporadic extra trainings on the topic	4<br>As in 2 + systematically extra trainings on the topic	5<br>As in 2 + systematically extra obligatory trainings on the topic, with innovative methods
2.6.a. How comprehensive is **treatment, beyond medication prescription for DM** also including measuring of BMI, waist circumference, BP measurements, cholesterol levels, renal function, screening for complications – foot exam, eye problems, macrovascular disease,	0<br><br>Not at all	1<br><br>Some elements	2<br><br>Most elements	3<br><br>All elements not consistently	4<br><br>All elements most of the time	5<br><br>Systematically all elements
2.6.b. How comprehensive is **treatment, beyond medication prescription for HT** also including measuring of BMI, BP measurements, palpation of pulsations, cholesterol measurement, ausculation of heart/carotides, screening for complications – urine exam, ECG,	0<br><br>Not at all	1<br><br>Some elements	2<br><br>Most elements	3<br><br>All elements not consistently	4<br><br>All elements most of the time	5<br><br>Systematically all elements

CV risk						
2.7.a. To what extent are **assessments undertaken in elderly** with DM? (functionality, memory, nutrition)	0<br>No questions about functionality memory or nutrition are asked	1<br>Some questions about functionality memory or nutrition are asked	2<br>In specific patients some of these assessments sometimes happen	3<br>In most patients some of the assessments happen	4<br>The complete assessment is routinely undertaken: functional, memory and nutrition	5<br>The complete assessment is routinely undertaken: functional, intervention plans are added, eg. Falling
2.7.b. To what extent are **assessments undertaken in elderly** with HT? (functionality, memory, nutrition)	0<br>No questions about functionality memory or nutrition are asked	1<br>Some questions about functionality memory or nutrition are asked	2<br>In specific patients some of these assessments sometimes happen	3<br>In most patients some of the assessments happen	4<br>The complete assessment is routinely undertaken: functional, memory and nutrition	5<br>The complete assessment is routinely undertaken: functional, intervention plans are added, eg. Falling
2.8.a. To what extent are **medication reviews undertaken in elderly** with DM? In order to avoid polypharmacia, hypoglycemia and renal dysfunction.	0<br>No medication reviews	1<br>For some patients GP does this on own initiative	2<br>GP does it routinely	3<br>GP does it routinely, sometimes asks advise of farmacist	4<br>This is sometimes done in multidisciplinary setting (farmacist)	5<br>This is routinely done in multidisciplinary setting (farmacist)
2.8.b. To what extent are **medication reviews undertaken in elderly** with HT? In order to avoid polypharmacia, hypoglycemia and renal dysfunction.	0<br>No medication reviews	1<br>For some patients GP does this on own initiative	2<br>GP does it routinely	3<br>GP does it routinely, sometimes asks advise of farmacist	4<br>This is sometimes done in multidisciplinary setting (farmacist)	5<br>This is routinely done in multidisciplinary setting (farmacist)
Element 3: **Health education and counselling to patients with DM and/or HT by non-physician care providers**<br>*Non-physician: other than doctors*	No or little implementation		Moderate implementation		Almost complete or full implementation	
3.1.a. To what extent, do patients with DM receive **information on how to reduce health risks** by non-physicians? *(information on chronic disease management and lifestyle support)*<br>[PROCESS]	0<br>Not at all	1<br>Only informal education is given	2<br>Within consultation by a non-physician	3<br>Structured individual education by a non-physician is scheduled for every patient	4<br>A multidisciplinary team provides structured education to groups of patients	5<br>Group sessions by a multidisciplinary team, the sessions are quality assured (reviewed) and make use of interactive techniques such as

						video, discussion
3.1.b. To what extent, do patients with HT receive **information on how to reduce health risks** by non-physicians? *(information on chronic disease management and lifestyle support)*<br>[PROCESS]	0<br><br>Not at all	1<br><br>Only informal education is given	2<br><br>Within consultation by a non-physician	3<br><br>Structured individual education by a non-physician is scheduled for every patient	4<br><br>A multidisciplinary team provides structured education to groups of patients	5<br><br>Group sessions by a multidisciplinary team, the sessions are quality assured (reviewed) and make use of interactive techniques such as video, discussion
3.2.a. To what extent, are patients **informed about the chronic condition** of DM bynon-physicians: including the expected course, expected complications, and effective strategies to prevent complications and manage symptoms? *(information on the prognosis)*<br>[PROCESS]	0<br><br>Not at all	1<br><br>Only informal education is given	2<br><br>Within consultation a non-physician	3<br><br>Structured individual education by a non-physician is scheduled for every patient	4<br><br>A multidisciplinary team provides structured education to groups of patients	5<br><br>Group sessions by a multidisciplinary team, the sessions are quality assured (reviewed) and make use of interactive technologies.
3.2.b. To what extent, are patients **informed about the chronic condition** of HT by non-physicians: including the expected course, expected complications, and effective strategies to prevent complications and manage symptoms? *(information on the prognosis)*<br>[PROCESS]	0<br><br>Not at all	1<br><br>Only informal education is given	2<br><br>Within consultation a non-physician	3<br><br>Structured individual education by a non-physician is scheduled for every patient	4<br><br>A multidisciplinary team provides structured education to groups of patients	5<br><br>Group sessions by a multidisciplinary team, the sessions are quality assured (reviewed) and make use of interactive technologies.
3.3.a. To what extent, ares **trained to provide health education and counselling** to patients with DM?<br>[STRUCTURE/EDUCATION]	0<br>Not at all	1<br>On the job training or when services started	2<br>Part of formal education to obtain certificate needed to do the job	3<br>as in 2 + sporadic extra trainings on the topic	4<br>As in 2 + systematically extra trainings on the topic	5<br>As in 2 + systematically extra obligatory trainings on the topic, with innovative methods
3.3.b. To what extent, are non-physicians **trained to provide health education and counselling** to patients with HT?	0<br>Not at all	1<br>On the job training or when services started	2<br>Part of formal education to obtain certificate needed	3<br>as in 2 + sporadic extra trainings on the topic	4<br>As in 2 + systematically extra trainings on the	5<br>As in 2 + systematically extra obligatory trainings on

[STRUCTURE/EDUCATION]			to do the job		topic	the topic, with innovative methods
3.4.a. To what extent, are **health education or counselling materials** accessible to non-physicians for DM?<br>[STRUCTURE]	0<br><br>Not available at all	1<br><br>Available (some materials)	2<br><br>All available but not accessible	3<br><br>Partially accessible to all necessary materials	4<br><br>Fully accessible to almost all necessary materials	5<br><br>Fully accessible to all necessary materials
3.4.b. To what extent, are **health education or counselling materials** accessible to non-physicians for HT?<br>[STRUCTURE]	0<br><br>Not available at all	1<br><br>Available (some materials)	2<br><br>All available but not accessible	3<br><br>Partially accessible to all necessary materials	4<br><br>Fully accessible to almost all necessary materials	5<br><br>Fully accessible to all necessary materials
Element 4: **Self-management support to patients and their informed caregivers with tools for adherence and monitoring**<br>*Self-management support: supporting patients to self-manage their conditions (practice and reinforce)*	No or little implementation		Moderate implementation		Almost complete or full implementation	
4.1.a. To what extent, are patients offered **self-management training** for DM (for example, to improve adherence to medications, proper nutrition, having self-monitoring tools at home, consistent exercise, tobacco cessation, and maintain other healthy behaviours)?<br>[PROCESS]	0<br><br>Not at all	1<br><br>Little offer (only one component of the list) to few patients	2<br><br>Limited offer to few patients	3<br><br>Limited offer to most patients	4<br><br>Offer all the mentioned to most patients	5<br><br>Offer all the mentioned to every patient
4.1.b. To what extent, are patients offered **self-management training** for HT (for example, to improve adherence to medications, proper nutrition, having self-monitoring tools at home, consistent exercise, tobacco cessation, and maintain other healthy behaviours)?<br>[PROCESS]	0<br><br>Not at all	1<br><br>Little offer (only one component of the list) to few patients	2<br><br>Limited offer to few patients	3<br><br>Limited offer to most patients	4<br><br>Offer all the mentioned to most patients	5<br><br>Offer all the mentioned to every patient
4.2.a. To what extent, do health	0	1	2	3	4	5

care staff or community health workers **support patients' self-management** efforts **on a continuous basis for DM?** [PROCESS]	Not at all	Only once when the service starts	In most visits, but no use of telephone/apps	Once a year via telephone or email	Once per quarter via telephone call or email	On every visit and supported with commonly used apps
4.2.b. To what extent, do health care staff or community health workers **support patients' self-management** efforts **on a continuous basis for HT?** [PROCESS]	0<br><br>Not at all	1<br><br>Only once when the service starts	2<br><br>In most visits, but no use of telephone/apps	3<br><br>Once a year via telephone or email	4<br><br>Once per quarter via telephone call or email	5<br><br>On every visit and supported with commonly used apps
4.3. To what extent, are health care staff or community health workers **competent to perform self-management training?** [STRUCTURE/EDUCATION]	0<br><br>Not at all	1<br><br>Know but cannot perform (not confidence or lack of equipment or materials	2<br><br>Can perform with guidance from others	3<br><br>Can perform limited training lessons	4<br><br>Can perform almost all the training lessons	5<br><br>Can perform all the training lessons
4.4.a. To what extent, does the patient have **access to material for self-monitoring for DM**, for instance, glucose meter/ glucose test strips.	**0**<br><br>Not exist	**1**<br><br>Exist in theory but access for patients is not organised	**2**<br><br>Access for some patients to all materials needed but refills lack	**3**<br><br>Access for some patients to all materials needed included refills (strips, lancets)	**4**<br><br>Well-organised<br><br>Access for all patients to all materials needed but refills lack	**5**<br><br>Well-organised<br><br>Access for all patients to all materials needed included refills (strips, lancets)
4.4.b. To what extent, does the patient have **access to material for self-monitoring for HT**, for instance, blood pressure meter	**0**<br><br>Not exist	**1**<br><br>Exist in theory but access for patients is not organised	**2**<br><br>Access for some patients to all materials needed but refills lack	**3**<br><br>Access for some patients to all materials needed included refills (strips, lancets)	**4**<br><br>Well-organised<br><br>Access for all patients to all materials needed but refills lack	**5**<br><br>Well-organised<br><br>Access for all patients to all materials needed included refills (bateries)
4.5.a1. To what extent are **informal caregivers** in the self-management PROCESSs for DM?<br><br>(i.e. family, friend, relative,	**0**<br><br>Not exist	**1**<br><br>Occasionally involved but no health knowledge	**2**<br><br>Occasionally involved but limited health knowledge	**3**<br><br>Fully involved with limited health knowledge	**4**<br><br>Fully involved with full knowledge but haven't received	**5**<br><br>Fully involved with full knowledge and supporting materials

neighbour)					any supporting materials	
4.5.a2. To what extent are community workers involved in the self-management PROCESSs for DM?<br><br>(i.e.volunteer health support group and peer educator - it must be informal not part of health care system)	**0**<br><br>Not exist	**1**<br><br>Occasionally involved but no health knowledge	**2**<br><br>Occasionally involved but limited health knowledge	**3**<br><br>Fully involved with limited health knowledge	**4**<br><br>Fully involved with full knowledge but haven't received any supporting materials	**5**<br><br>Fully involved with full knowledge and supporting materials
4.5.b1. To what extent are **informal caregivers** in the self-management PROCESSs for HT?<br><br>(i.e. family, friend, relative, neighbour)	**0**<br><br>Not exist	**1**<br><br>Occasionally involved but no health knowledge	**2**<br><br>Occasionally involved but limited health knowledge	**3**<br><br>Fully involved with limited health knowledge	**4**<br><br>Fully involved with full knowledge but haven't received any supporting materials	**5**<br><br>Fully involved with full knowledge and supporting materials
4.5.b2. To what extent are community workers involved in the self-management PROCESSs for HT?<br><br>(i.e.volunteer health support group and peer educator - it must be informal not part of health care system)	**0**<br><br>Not exist	**1**<br><br>Occasionally involved but no health knowledge	**2**<br><br>Occasionally involved but limited health knowledge	**3**<br><br>Fully involved with limited health knowledge	**4**<br><br>Fully involved with full knowledge but haven't received any supporting materials	**5**<br><br>Fully involved with full knowledge and supporting materials
4.6. Are the **concerns of patients and families** addressed?	0<br>Not at all	1<br>Is not consistently done	2<br>Is provided for specific patients and families through referral	3<br>Is provided for specific patients and families in primary care	4<br>Is encouraged, and peer support, groups and mentoring programs are available	5<br>Is an integral part of primary care and includes systematic assessment and routine involvement in peer support, groups or mentoring programs
4.7. Are **patient treatment plans** used, are they agreed with	0<br>patient plans	1<br>patient treatment	2<br>pt treatment plans	3<br>are established	4<br>are established	5<br>are established

patients, reviewed and written down?	are not expected	plans only sometimes written down just	are achieved through a standardized approach for majority of patients	collaboratively with patient and include clinical goals	collaboratively with patient and include clinical goals as well as self management	collaboratively with patient and include clinical goals as well as self management. Follow-up occurs and guides care
Element 5: **Structured collaboration between health care workers, community actors, and patients and caregivers**	No or little implementation		Moderate implementation		Almost complete or full implementation	
5.1.a. To what extent, is there an identified **"care coordinator"** who serves as the overseer and director of a patient's care, ensuring that efforts of all involved health care workers, community actors, and patients and caregivers are integrated and coordinated for DM? [STRUCTURE]	0<br><br>Not exist	1<br><br>Exist but not active	2<br><br>Exist and active only when triggered	3<br><br>Exist and active occasionally	4<br><br>Exist and active but not structured	5<br><br>Exist and active and structured
5.1.b. To what extent, is there an identified **"care coordinator"** who serves as the overseer and director of a patient's care, ensuring that efforts of all involved health care workers, community actors, and patients and caregivers are integrated and coordinated for HT? [STRUCTURE]	0<br><br>Not exist	1<br><br>Exist but not active	2<br><br>Exist and active only when triggered	3<br><br>Exist and active occasionally	4<br><br>Exist and active but not structured	5<br><br>Exist and active and structured
5.2.a. To what extent, do the **health care organization and the community** have **complementary functions**, that is, community organizations fill gaps in services that are not provided in formal health care for DM? [STRUCTURE]	0<br><br>Not at all	1<br><br>Community effort exists but not relevant to the gap	2<br><br>Community effort exists and relevant but unable to fill the gaps	3<br><br>Community exists and filling limited gaps	4<br><br>Community exists and almost filling the gaps	5<br><br>Community exists and filling all the gaps
5.2.b. To what extent, do the **health care organization and the**	0	1	2	3	4	5

**community** have **complementary functions**, that is, community organizations fill gaps in services that are not provided in formal health care for HT [STRUCTURE]	Not at all	Community effort exists but not relevant to the gap	Community effort exists and relevant but unable to fill the gaps	Community exists and filling limited gaps	Community exists and almost filling the gaps	Community exists and filling all the gaps
5.3.a. To what extent, are **referral practices** systematically organised for DM? [STRUCTURE]	0<br><br>Not at all	1<br><br>Limited Referral and only one direction	2<br><br>Referral and only one direction	3<br><br>Referral organised informally for two directions	4<br><br>Limited Referral organised systematically for two directions	5<br><br>Referral structured systematically for two directions
5.3.b. To what extent, are **referral practices** systematically organised for HT? [STRUCTURE]	0<br><br>Not at all	1<br><br>Limited Referral and only one direction	2<br><br>Referral and only one direction	3<br><br>Referral organised informally for two directions	4<br><br>Limited Referral organised systematically for two directions	5<br><br>Referral organised systematically for two directions
5.4.a. To what extent does **cooperation between health care workers and other professionals and community actors** occur for DM?	0<br><br>No cooperation	1<br><br>Little cooperation without regular discussions	2<br><br>Moderate cooperation within the team with regular discussions	3<br><br>Full cooperation within teams, but not across	4<br><br>Cooperation within teams and across teams to some external team	5<br><br>Multi-disciplinary cooperation across all levels
5.4.b. To what extent does **cooperation between health care workers and other professionals and community actors** occur for HT?	0<br><br>No cooperation	1<br><br>Little cooperation without regular discussions	2<br><br>Moderate cooperation within the team with regular discussions	3<br><br>Full cooperation within teams, but not across	4<br><br>Cooperation within teams and across teams to some external team	5<br><br>Multi-disciplinary cooperation across all levels
5.5.a. To what extent is the traditional **hierarchy flattened** and moved away from physician dominated models for DM?	0<br>Specialists are dominating, also in first-line	1<br>For some patients specialists are central, for other general practitioners	2<br>General practitioners are central, there are no other HCW	3<br>General practitioners are central, there are no other HCW play minor role	4<br>General practitioners are central, other HCW play big role	5<br>HCW with special training in chronic care is central in the multidisciplinary team
5.5.b. To what extent is the traditional hierarchy flattened and moved away from physician dominated models for HT?	0<br>Specialists are dominating, also in first-line	1<br>For some patients specialists are central, for other general	2<br>General practitioners are central, there are no other HCW	3<br>General practitioners are central, there are no other HCW play minor role	4<br>General practitioners are central, other HCW play big role	5<br>HCW with special training in chronic care is central in the multidisciplinary team

		practitioners				
Element 6: **Questions regarding organisation of care, delivery system design and clinical information systems**	No or little implementation		Moderate implementation		Almost complete or full implementation	
6.1 To what extent are ongoing **quality improvement** routine activities among health care workers organised?	0<br>No quality improvement	1<br>New rules to improve quality are sometimes set from management	2<br>When a problem pops up a quality improvement activity is sometimes undertaken	3<br>When a problem pops up a quality improvement activity is often undertaken	4<br>Is a routinely PROCESSs but results from previous round are often not taken into account	5<br>Is a routinely PROCESSs and results from previous round are taken into account
6.2 To what extent do **information systems** gather and organise data about epidemiology, treatment, and health care outcomes?	0<br>There is no registry	1<br>There is a registry but is not used for treatment purpose	2<br>The registry includes name, diagnosis, contact info and date of last contact	3<br>The registry includes name, diagnosis, contact info, date of last contact, treatment and outcomes	4<br>The registry allows queries to sort subpopulations by clinical priorities	5<br>The registry is tied to guidelines which provide prompts and reminders about needed services
6.3 To what extent is **information about relevant subgroups** of patients needing services available?	0<br>is not available	1<br>can not be obtained by primary care provider	2<br>can only be obtained with special efforts or additional programming and is incomplete	3<br>can only be obtained with special efforts or additional programming	4<br>can be obtained upon request but is not routinely available	5<br>is provided routinely to providers to help them deliver planned care and is used for this purpose
6.4 To what extent do information systems serve a **reminder function** for patient specific prevention and follow-up services (e.g. to identify patients' needs, to follow-up and plan care, to monitor responses to treatment, and to assess health outcomes)?	0<br>No Information system	1<br>No reminder function	2<br>Reminders include general notification of the existence of a chronic illness, but does not describe needed services at time of encounter	3<br>Reminders describe needed services at time of encounter, based on general guideline, not patient-specific	4<br>Includes specific information for the patient at the time of individual patient encounters	5<br>Includes specific information for the team about adherence to patient care plan at the time of individual patient encounters
6.5 To what extent is **feedback about the performance provided to the team and its members**?	0<br>Not available	1<br>Non-specific to the team	2<br>Infrequent intervals and not delivered to	3<br>Frequent intervals but not specific for the	4<br>Occurs at frequent enough intervals to	5<br>Timely, specific to the team, routine and

			the team (teams if they want they search for the information)	team and impersonally delivered (just common reports)	monitor performance and is specific to the team’s population	personally deliveredby a respected opinion leader
6.6 To what extent is an **appointment system** with planned visits used?	0<br>No appointment system	1<br>Used to schedule acute care visits, follow-up and preventive visits	2<br>Appointment system assures scheduled follow-up with chronically ill patients, but some pts escape the system	3<br>Appointment system assures scheduled follow-up with chronically ill patients	4<br>Appointment system are flexible and can accommodate innovations such as customized visit length or group visits	5<br>Appointment system includes organization of care that facilitates the patient seeing multiple providers in a single visit.
