# Supplementary material for: Implementation of Integrated Primary Care for Patients with Diabetes and Hypertension: A Case from Slovenia
Source: Int J Integr Care. 2021 Sep 28;21(3):15. doi: 10.5334/ijic.5637 (PMC8485865; doi:10.5334/ijic.5637)
Supplement: Appendix 2. — Assessed implementation of integrated care for patients with diabetes and/or hypertension according to individual health care organisation. [file ijic-21-3-5637-s2.pdf]

Appendix 2: Assessed implementation of integrated care for patients with diabetes and/or hypertension according to individual health care organisation

	E1 Mean (SD)	E2 Mean (SD)	E3 Mean (SD)	E4 Mean (SD)	E5 Mean (SD)	E6 Mean (SD)	Overall† Mean (min, max)
Rural family practice team 1	5.0 (0.0)	4.1 (0.8)	4.0 (0.8)	2.7 (1.7)	2.6 (1.6)	4.0 (2.0)	3.7 (2.6, 5.0)
Rural family practice team 2	4.8 (0.5)	3.8 (1.4)	4.3 (0.5)	3.1 (1.7)	3.6 (2.1)	3.2 (1.8)	3.8 (3.1, 4.8)
Urban family practice team 1	5.0 (0.0)	4.1 (0.8)	4.0 (0.8)	2.2 (1.9)	2.6 (1.6)	4.0 (2.0)	3.7 (2.2, 5.0)
Urban family practice team 2	5.0 (0.0)	4.1 (0.9)	4.0 (0.8)	2.2 (1.8)	3.3 (2.0)	4.2 (1.6)	3.8 (2.2, 5.0)
Urban family practice team 3	5.0 (0.0)	3.7 (1.5)	3.5 (0.8)	2.2 (1.9)	3.1 (1.9)	3.8 (1.6)	3.6 (2.2, 5.0)
Urban family practice team 4	5.0 (0.0)	3.7 (1.3)	5.0 (0.0)	2.2 (1.9)	3.2 (2.0)	4.2 (1.6)	3.9 (2.2, 5.0)
Urban family	5.0	4.1	4.5	2.3	3.1	3.7	3.8 (2.3,

practice team 5	(0.0)	(1.0)	(0.9)	(1.9)	(1.9)	(1.8)	5.0)
Urban family practice team 6	5.0 (0.0)	4.0 (0.9)	4.5 (0.9)	2.2 (1.9)	2.4 (2.0)	3.5 (2.0)	3.6 (2.2, 5.0)
Urban family practice team 7 (independent contractor)	5.0 (0.0)	3.9 (1.1)	4.3 (0.9)	2.2 (1.7)	2.9 (1.9)	3.3 (1.5)	3.6 (2.2, 5.0)
Urban family practice team 8 (independent contractor)	5.0 (0.0)	4.0 (0.9)	4.0 (0.8)	2.5 (1.7)	3.4 (2.0)	2.8 (1.2)	3.6 (2.5, 5.0)

E1 – Identification

E2 – Treatment

E3 – Health Education

E4 – Self-management Support

E5 – Structured collaboration

E6 – Care organisation

SD = standard deviation

(†) Overall ICP score of a health organisation was assessed as a mean of scores for separate ICP elements.
